# Self-care and remote care during pregnancy: a new paradigm?

**DOI:** 10.1186/s12961-020-00627-4

**Published:** 2020-09-18

**Authors:** A. Metin Gülmezoglu, Anne Ammerdorffer, Manjulaa Narasimhan, Alyce N. Wilson, Joshua P. Vogel, Lale Say, Özge Tunçalp

**Affiliations:** 1 Concept Foundation, Geneva, Switzerland; 2 UNDP/UNFPA/UNICEF/WHO/World Bank Special Programme of Research, Development and Research Training in Human Reproduction, Department of Sexual and Reproductive Health and Research, World Health Organization, Geneva, Switzerland; 3 Maternal, Child and Adolescent Health Program, Burnet Institute, Melbourne, Australia

**Keywords:** Self-care, remote care, antenatal care, pregnancy

## Abstract

Self-care interventions and remote care offer innovative and equitable ways to strengthen access to sexual and reproductive health services. Self-isolation during COVID-19 provided the opportunity for obstetric facilities and healthcare providers to integrate and increase the usage of interventions for self-care and remote care for pregnant women and to improve the quality of care overall.

## Commentary

Self-care is defined by WHO as “*the ability of individuals, families and communities to promote health, prevent disease, maintain health, and to cope with illness and disability with or without the support of a healthcare provider*” [1]. Self-care interventions offer innovative and equitable ways to strengthen access to sexual and reproductive health services, especially in rural and low-resource settings experiencing provider shortages [2]. Self-care interventions can potentially reduce the cost of care by reducing travel to facilities and time off work. It is also likely that people and health workers will be more at ease with self-care options with the increasing availability of digital tools and technologies [3].

In 2016, WHO published 49 antenatal care (ANC) recommendations for a positive pregnancy experience [4]. In this guideline, WHO recommended a minimum of eight contacts between the pregnant woman and the health system and emphasised the importance of supportive communication and information exchange. The increased number of contacts were aimed at improving the likelihood of diagnosing asymptomatic conditions that may pose a risk to the health of the mother and the fetus. The main content of additional contacts includes checking blood pressure and urine, ensuring that the fetal heart rate is detected and that there are no obvious abnormalities. Perhaps more importantly, these added contacts allow caregivers to respond to the pregnant woman’s questions, provide counselling on healthy behaviours and discuss key postpartum issues such as breastfeeding and contraception.

In 2019, WHO published the first consolidated ‘living’ guideline on self-care health interventions, which included ANC interventions for nausea and vomiting, heartburn, leg cramps and constipation [1]. Additional opportunities that self- and remote-care interventions present to pregnancy care will be further developed in the context of this ‘living’ guideline.

Both self-care and remote care are feasible for pregnancy care. Several interventions can be accessed, used and administered by women themselves such as self-monitoring of blood pressure and urine testing. Remote care uses information technology to gather and exchange data outside of a facility. This can provide much needed health information and counselling as well as providing guidance on self-care activities. These two concepts can change and improve the way ANC is provided, leading to a fundamental and sustainable positive shift in how pregnancy care is provided.

There is emerging evidence that, taken together, facilities and healthcare administrators can successfully tailor the ANC provision regarding the number and frequency of in-person contacts suitable for their population and setting with self-care and remote care. Butler Tobah et al. [5] conducted a randomised controlled trial comparing standard ANC with a mixed-care system that incorporated remote care for blood pressure measurement, fetal heart rate assessment and access to an online community of pregnant women. They found higher satisfaction with care, less anxiety and no differences in health outcomes despite reduced in-person contact. It should be noted that the intervention was delivered to a relatively affluent population in the United States where standard care included 12 to 14 in-person contact visits – much higher than the eight contacts recommended by WHO. Nevertheless, it is indicative of the way that ANC provision may change in the next decade and suggests that these changes can have benefits. A recent systematic review by DeNicola et al. [6] on telehealth interventions, such as text messaging, online consultations and self-monitoring with messaging (among others), further demonstrated that remote-care interventions are effective in improving perinatal smoking cessation, breastfeeding and early access to medical abortion services as well as optimising use of high-risk obstetrical services.

The COVID-19 pandemic has led to a rapid redirection of health services as countries prepare and respond. Sexual and reproductive health services have been particularly affected – some countries have chosen to cease or restrict ANC or contraception programmes due to staff or resource limitations or to prevent COVID-19 transmission [7]. In WHO’s latest operational guidance for maintaining essential health services, one of the key actions identified is “*using available technologies and associated regulations to facilitate the shift of clinical encounters to digital platforms and to support self-care interventions wherever appropriate*” [8].

Some health facilities in the United States have successfully implemented antenatal remote care during COVID-19 to ensure safe and effective obstetric care. For example, obstetric facilities in Florida, Texas and New York reduced their in-person visits from 11 to 6, 13 to 9, and 11 to 6 respectively [9–11]. They developed their ANC schedules based on expert recommendations, established WHO and national guidelines, and the OB Nest model developed at Mayo Clinic for women experiencing low-risk pregnancies [12].

Despite the upheaval, lessons from self-isolation during COVID-19 provide an opportunity to increase interventions for self-care and remote care for pregnant women and to improve the quality of care overall. In addition to rethinking how routine ANC can be best provided, it offers strategies for future pandemics or other large-scale service disruptions. Digital interventions, both visual and audio, are likely to increase our options for self-care and remote care that can improve ANC provision and perhaps even have a positive impact on health outcomes and the women’s experience of care. The acute challenges of the pandemic can facilitate an accelerated response to these innovations to improve the quality of care and make ANC more woman, family and community friendly.

## Data Availability

Not applicable.

## Abbreviation

ANC: antenatal care
