# Correction to: Self-care and remote care during pregnancy: a new paradigm?

**DOI:** 10.1186/s12961-020-00641-6

**Published:** 2020-10-12

**Authors:** A. Metin Gülmezoglu, Anne Ammerdorffer, Manjulaa Narasimhan, Alyce N. Wilson, Joshua P. Vogel, Lale Say, Özge Tunçalp

**Affiliations:** 1 Concept Foundation, Geneva, Switzerland; 2 UNDP/UNFPA/UNICEF/WHO/ World Bank Special Programme of Research, Development and Research Training in Human Reproduction, Department of Sexual and Reproductive Health and Research, World Health Organization, Geneva, Switzerland; 3 Maternal, Child and Adolescent Health Program, Burnet Institute, Melbourne, Australia

**Correction to: Health Res Policy Sys 18, 107 (2020)**

**https://doi.org/10.1186/s12961-020-00627-4**

It was highlighted that the original article [1] contained a numerical error on the second page of the article. This Correction article shows the correct and incorrect sentence. The original article has been updated.

**Correct sentence**

It should be noted that the intervention was delivered to a relatively affluent population in the United States where standard care included 12 to 14 in-person contact visits – much higher than the 8 contacts recommended by WHO.

**Incorrect sentence**

It should be noted that the intervention was delivered to a relatively affluent population in the United States where standard care included 12 to 14 in-person contact visits – much higher than the 88 contacts recommended by WHO.
